# High-Yield Production of SiV-Doped Nanodiamonds for Spectroscopy and Sensing Applications

**DOI:** 10.1021/acsanm.4c04676

**Published:** 2024-10-25

**Authors:** Alexander Kromka, Marián Varga, Kateřina Aubrechtová Dragounová, Oleg Babčenko, Rene Pfeifer, Assegid M. Flatae, Florian Sledz, Farzana Akther, Mario Agio, Štěpán Potocký, Štěpán Stehlík

**Affiliations:** † Institute of Physics, Czech Academy of Sciences, Cukrovarnická 10/112, Prague 6 162 00, Czech Republic; ‡ Institute of Electrical Engineering, Slovak Academy of Sciences, Dúbravská Cesta 9, Bratislava 841 04, Slovakia; § Faculty of Nuclear Sciences and Physical Engineering, Czech Technical University in Prague, Břehová 7, 115 19, Prague 1, Prague 6 162 00, Czech Republic; ∥ Laboratory of Nano-Optics and Cμ, University of Siegen, Walter-Flex-Str. 3, Siegen 57072, Germany; ⊥ National Institute of Optics (INO−CNR), Largo Enrico Fermi 6, Florence 50125, Italy

**Keywords:** porous diamond films, molten salt thermal etching, SiV-doped nanodiamonds, dynamic light scattering, transmission electron microscopy, photoluminescence, temperature-resolved spectroscopy

## Abstract

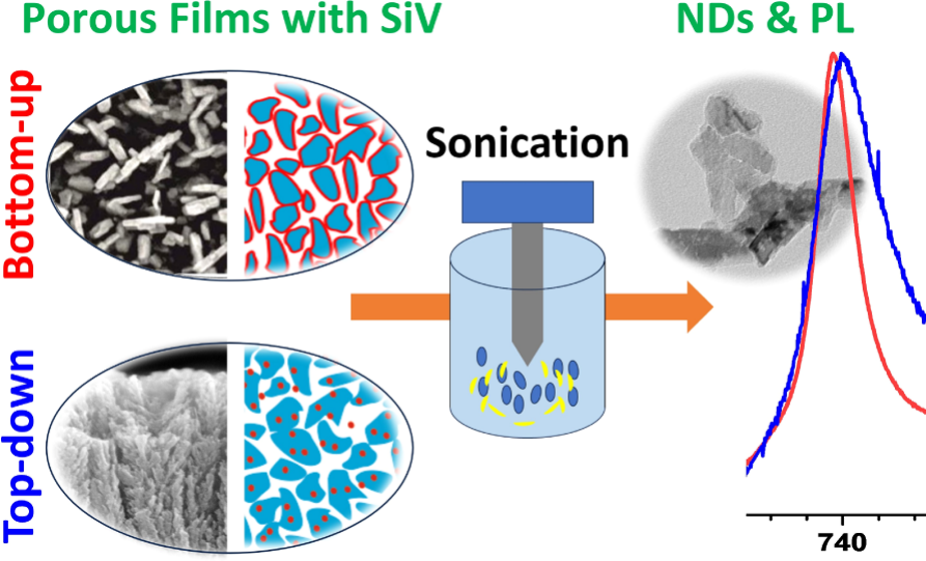

Nanodiamonds (NDs) containing optically active centers have gained significant relevance as the material of choice for biological, optoelectronic, and quantum applications. However, current production methods lag behind their real needs. This study introduces two CVD-based approaches for fabricating NDs with optically active silicon-vacancy (SiV) color centers: bottom-up (BU) and top-down (TD) methods. The BU approach generates nanoporous diamond films with a core–shell structure, while the TD method employs molten-salt thermal etching to create uniform porous structures from nanocrystalline diamond films. Comprehensive characterization using advanced techniques revealed distinct morphologies and optical properties for each approach. The BU method yielded higher-quality diamond phases with top-surface incorporation of SiV centers, while the TD method demonstrated efficient nondiamond phase removal. Ultrasonic disintegration of both porous films produced NDs ranging from 40 to 500 nm, with unique morphologies characteristic of each approach. Photoluminescence measurements confirmed SiV centers (738 nm) in all NDs, exhibiting sensitivity to surface terminations, particularly in BU samples. Temperature-resolved spectroscopy shows the potential of the fabricated NDs for nano thermometry over a wide range of temperatures up to 100 °C. The zero-phonon line shows 0.022 ± 0.003 nm/K sensitivity, while the line width exhibits 0.068 ± 0.004 nm/K broadening. The presented BU and TD methods offer significant advantages over existing techniques, including streamlined production processes, high-yield ND synthesis with tailored properties, and the potential for scalable, cost-effective manufacturing.

## Introduction

Diamond is recognized as a prospective multifunctional material for various multidisciplinary uses. Today, the synthesis of diamond films by the chemical vapor deposition (CVD) process has become a routine in laboratories and companies worldwide.^1^ Doping of diamond with foreign atoms (nitrogen, silicon, germanium, tin, etc.) can result in the formation of photoluminescent optical centers making them preferable for photonic applications, quantum technologies, optically active markers for life science and sensors, etc.^2−6^ From the large family of photoluminescent optical centers (>400), silicon-vacancy (SiV) has received lot of attention due to stable and nonblinking photoluminescence (PL) with a sharp emission band at the near-infrared spectral region with the zero-phonon line at 738 nm. SiV naturally appears in the diamond films as a result of the self-doping process when the Si substrates are used or when the CVD growth is performed with the presence of Si, SiC, or quartz solid sources.^7−9^ Fully controllable doping profiles with Si atoms can be realized by adding Si-containing gases (e.g., silane and tetramethylsilane) to the CVD growth mixture.^10−12^ Finally, ion implantation can also be used to incorporate Si into the diamond crystal structure with excellent spatial control.^13^

Instead of the SiV diamond films, the diamond nanosized form known as nanodiamonds (NDs) is additionally advantageous^14^ and highly demanded in bioimaging and labeling.^12,15^ So far, only a few techniques have proven the ability to produce NDs with SiV centers such as high-pressure high-temperature (HPHT) synthesis^16−18^ and even detonation synthesis.^19^ The CVD technology, in principle, offers great control over diamond doping in terms of the precursor quality and its concentration, as well as over the morphology and phase composition of the diamond film. However, the apparent limitation of CVD technology for the preparation of NDs is the compactness of conventional CVD layers and very energetic methods must be used to produce NDs.^20^ Several strategies can overcome this limitation. For example, by tuning the growth conditions of the CVD process toward the high power densities, 100–400 nm loose diamond particles with NV, SiV, and GeV centers were obtained.^21,22^ The compactness of CVD layers can also be overcome by introducing pores that would make the disintegration easier by using more gentle methods such as sonication. It has been recently shown that by using a special growth mode, a porous morphology of the nanocrystalline diamond (NCD) layer can be achieved by bottom-up (BU)^23−25^ or top-down (TD) approaches.^26−28^ Here, the porous morphology can be envisioned as a highly suitable material platform for NDs preparation thanks to its brittleness and easy breakability into individual crystalline nanograins. Such obtained NDs would utilize the excellent control of the CVD technique over the diamond properties, including controlled doping. Finally, one can envision a layer that has a high content of the sp^2^-C grain boundary phase which effectively separates the individual diamond nanocrystals. This is a common visualization of so-called ultra-nanocrystalline diamond (UNCD) layers. By using a proper treatment,^29,30^ the interparticle nondiamond phase could be selectively etched away and then the individual NDs could be obtained.

In this work, we establish the direct BU growth and the unique TD treatment of diamond thin films to achieve highly porous diamond structures that are used as the basis for the fabrication of NDs with SiV optical centers (SiV NDs). In particular, we developed the two proposed viable approaches for SiV NDs preparation and compared them from the perspective of the SiV optical properties, ND structure, size, and yield. Temperature-resolved spectroscopy is used to demonstrate that NDs can potentially be used for nano thermometry over a wide range of temperatures, exhibiting a linear red shift in the zero-phonon line and spectral broadening with increasing temperature.

## Experimental Section

### Material Fabrication and Processing

One-sided polished silicon (100) substrates of size 1 × 1 cm^2^ were used for CVD diamond growth. First, the Si substrates were cleaned in an ultrasonic bath (Transonic T490 DH, Elma, *f* = 40 kHz, 100% of 350 W) in isopropanol followed by rinsing with deionized (DI) water and then seeded in a detonation ND water-based suspension in the same ultrasonic bath according to the work of Kromka et. al.^31^ Porous diamond films were fabricated by either a BU or a TD approach.

### BU Approach

First, diamond films (thickness <500 nm) with a porous morphology were grown in the linear antenna pulsed microwave (MW) plasma system (AK 400, Roth & Rau).^32^ The MW power was supplied through two linear antennas by two MW generators (MX4000D, Muegge, 2.45 GHz) working at a pulse frequency of up to 500 Hz, and the MW power ON/OFF pulse cycle was set to 6:3 ms. A two-step CVD procedure with nominally “flat” starting diamond growth in the first step (hydrogen-rich gas mixture) and porous diamond growth in the second step (oxygen-rich gas mixture) was employed.^25^ The deposition parameters are summarized in Table S1.

**Scheme 1:**
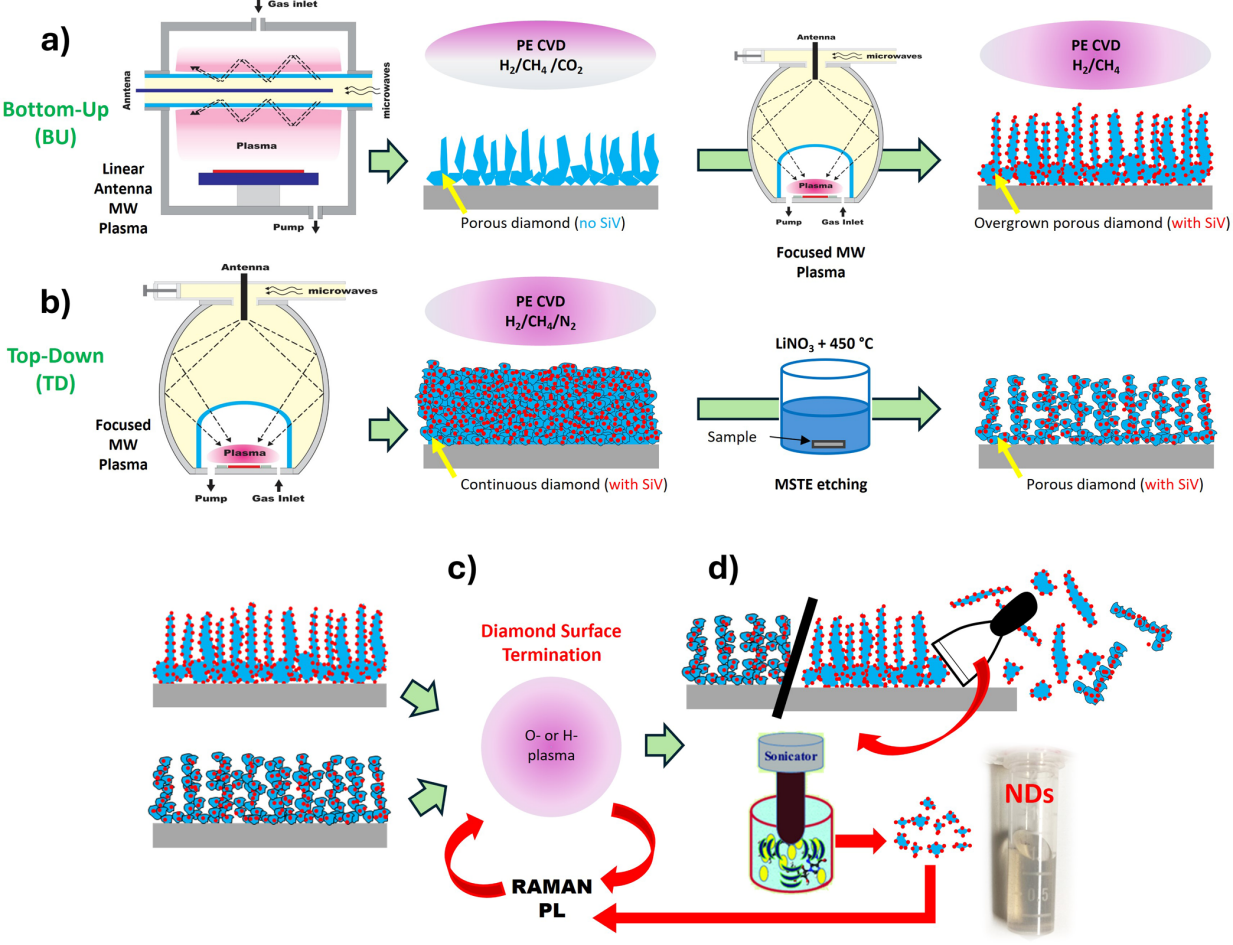
Schematic Representation of the (a) BU and (b) TDApproaches for O- and H-Terminated (c) Porous NCD with SiV Optical Centers Dispersed in Water and Disintegrated by an Ultrasonic Horn (d) into Nanodiamonds

Then, the surface of the initial porous diamond film was overgrown with a thin layer (i.e., labeled as an overgrown layer) of Si-doped diamond in a focused MW plasma system,^33^ clean (nonseeded) Si wafer pieces placed near the substrates were used as a solid source of silicon, and the deposition process parameters are summarized in Table S2. The BU fabrication approach is shown in Scheme 1a.

### TD Approach

In the TD approach, first, the NCD films with a thickness of 5.5 μm were grown on Si substrates in the focused MW plasma system (P6, Aixtron). During the CVD growth, clean (nonseeded) Si wafer pieces placed near the substrates were used as a solid source of silicon. The deposition process parameters for the growth of NCD films with SiV centers are summarized in Table S3.

To turn the continuous NCD films into porous ones, they were treated with a molten salt thermal etching (MSTE) process.^30^ First, an aqueous solution of 1.0 M LiNO_3_ (99.5%, Carl Roth) was prepared by dissolving 1.767 g of pure substance in 25 mL of laboratory-grade DI water. Then, 20 μL of the 1 M of LiNO_3_ solution was deposited on the NCD samples by a drop-casting technique. The samples covered with LiNO_3_ were treated at 450 °C for 5 h under an ambient atmosphere in a laboratory furnace. Then, the formed porous NCD films were cleaned in a solution of 1.0 M HCl (35%, Penta Chemicals) in an ultrasonic bath (Transonic T 490 DH, Elma, 40 kHz) for 5 min, followed by DI water rinse and dried by nitrogen. The TD fabrication approach is shown in Scheme 1b.

### Surface Termination of Porous Diamond Films

To investigate the effect of surface termination on PL emission of SiV color centers, the BU and TD porous diamond films were treated in either hydrogen or oxygen plasma. As the starting point, the BU and TD porous diamond samples were first treated by the hydrogen plasma in the focused MW plasma to achieve a hydrogen-terminated surface for both porous films, Table S4. The oxygen surface termination of the BU and TD porous diamond samples (primary hydrogen terminated) was done using radiofrequency oxygen plasma (Diener Electronic, FEMTO, 13.56 MHz), Table S5.

### Fabrication of Nanodiamonds

The DI water-based SiV-containing ND colloids were prepared from BU or TD porous diamond samples. In the case of BU samples, the diamond film was first mechanically peeled off of the substrate. Then, the peeled-off material was dispersed in 2 mL of DI water and treated by an ultrasonic horn (Hielscher UP200S, 24 kHz) at a power of 200 W lasting for 1 h. In the case of TD samples, the SiV NDs colloid was prepared by dispersing of porous film in 1 mL of DI water and then underwent the same ultrasonic procedure as mentioned above.

### Material Characterization

The scanning electron microscopy (SEM) images of BU and TD porous diamond films were acquired at 10 kV under 0° (top-view) and 90° (cross-section view) in the regime of secondary electrons (e-line system, Raith GmbH, Germany).

The Raman and PL measurements were acquired using an InVia Reflex Raman microscope (Renishaw) equipped with a laser with an excitation wavelength of 442 nm (Dual Wavelength HeCd, model IK5651R-G, Kimmon Koha) and air-cooled CCD camera. The samples were excited by a continuous wave laser (the power of 2.6 mW) focused to a spot of 1 μm on the sample in a perpendicular direction to the sample plane using 100× objective Leica with NA = 0.9. Raman spectra of BU and TD porous diamond samples were accumulated for 30 s, and the spectra were sampled 5 times. PL spectra were accumulated for 10 s and sampled 3 times. To quantify the portion of diamond to nondiamond carbon phases, the baseline inferred by asymmetric least-squares smoothing method (ALS, asymmetric factor 5 × 10^–5^, threshold 0.005, smoothing factor of 4, and 20 iterations) was subtracted from Raman spectra. After that, all spectra were normalized to the intensity of the diamond peak and fitted by the Lorenz and Gaussian curves. The PL emission spectra were corrected for the spectral dependence of the apparatus response. To eliminate the effect of PL background on SiV center PL, the PL background was plotted with the B-spline interpolation method (10 anchor points found with the first and second derivation method) and subtracted from the total spectrum. After that, the integral intensity of the SiV zero-phonon line maximum around 13,540 cm^–1^ (738.55 nm) as an integrated area under the curve was determined. In the case of SiV NDs made from BU and TD porous diamond samples, the prepared colloids were drop-cast on a flat Si substrate and dried. This procedure was repeated multiple times to ensure a sufficient sample amount for detection. After that, samples were analyzed using the aforementioned Raman and PL measurement setup.

Temperature-resolved spectroscopy was performed using a custom-built temperature-controlled sample stage. The sample was excited by 532 nm (Thorlabs CPS 532) and 638 nm (PicoQuant, PDL 800-D, LDH-D-C-640B) laser sources to excite SiV color centers and also check the possible presence of other defects (like nitrogen-vacancy (NV) center) incorporated during the CVD growth process. The emission from the sample was collected via a long working distance microscope objective (Zeiss LD C Epiplan-APOCHROMAT 50×, 0.6 NA). Here, we want to point out that the drop-casting procedure was performed only once due to the ability to detect individual scattered particles. All temperature-dependent optical measurements are conducted on ensembles of NDs with different morphologies and size distribution.

Dynamic light scattering (DLS) and zeta-potential (ζ) measurements of SiV NDs were performed on a Malvern Zetasizer Nano ZS equipped with a helium–neon laser (633 nm), and the scattering angle was 173°. The refractive index of bulk diamond (2.4) and the viscosity of pure water (1.020 mPa s) were used to convert the measured intensity/size distributions to volume/size distributions.

The size and shape of SiV ND particles were analyzed by a transmission electron microscope FEI Tecnai G2 20 with a LaB6 cathode at an acceleration voltage of 200 kV. The transmission electron microscope was equipped with a CCD camera, an Olympus Veleta.

## Results and Discussion

### Surface Morphology of BU and TD Porous Diamond Films

**Figure 1:**
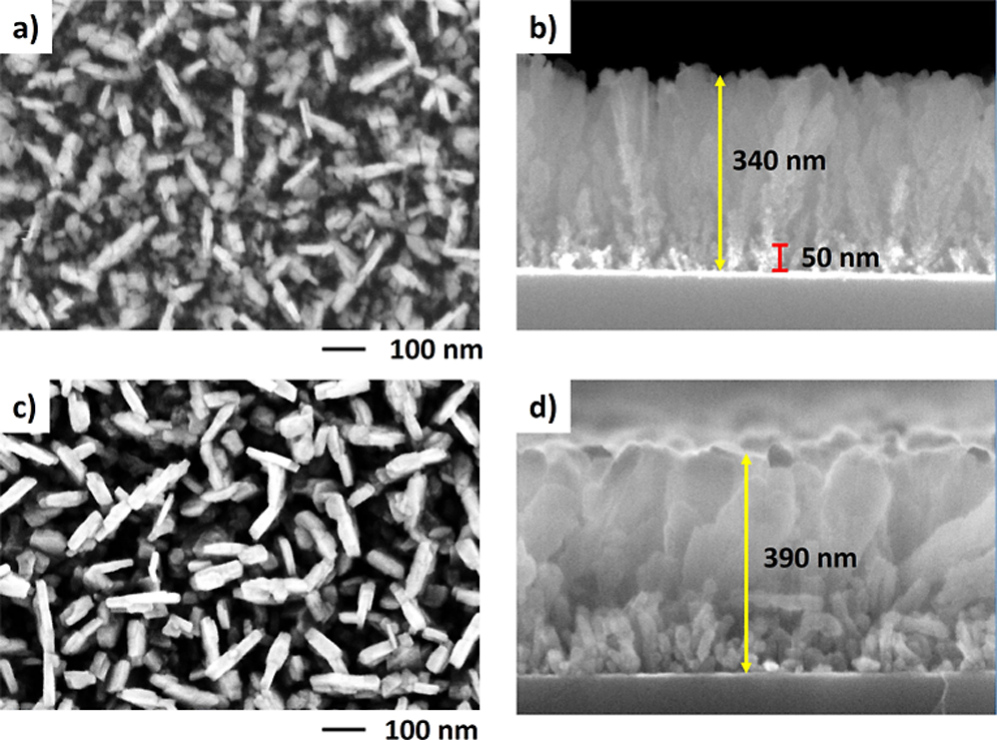
Surface morphology of the BU sample—top and cross-sectional SEM images of the initial porous diamond film without SiV centers (a,b) and the porous diamond film overgrown with a thin diamond film containing SiV centers (c,d).

Representative top- and cross-sectional SEM images of porous diamond films (BU approach) are shown in Figure 1. The initial porous diamond film consists of randomly distributed dendrite-like diamond nanograins with a thickness ranging from 10 to 30 nm and lengths varying between 50 and 200 nm (Figure 1a,b). The maximum distance between these nanograins does not exceed 100 nm, indicating the maximum size of the created nanopores. The total thickness of the diamond film is approximately 340 nm. Such unconventional growth of dendrite-like diamonds has previously been reported only for a pulsed MW plasma system with a linear antenna arrangement using a low working gas pressure in combination with a high CO_2_ content in the gas mixture.^25,32^

In the BU approach, the starting layer was formed during the first growth step, known as the “hydrogen-rich” growth regime (Table S1). This layer exhibits a flat and compact morphology, reaching a thickness of approximately 50 nm before the second growth regime is turned on. The starting layer improved process reliability by minimizing unwanted etching of the seeding layer in the oxygen-rich gas mixture used in the second growth regime, which resulted in better and homogeneous development of the subsequent porous layer. The second growth regime is characterized by a high CO_2_ content, resulting in a high amount of oxygen species (–O, –OH). These species preferentially attack less stable defects in the diamond crystals, enhancing the surface renucleation process and ultimately leading to the formation of porous morphology. The strong etching effect is evident in the cross-sectional SEM image (Figure 1b), where the lighter bottom area indicates partial etching of the primary layer during early formation of the porous layer. However, the high CO_2_ content in the gas mixture suppresses the formation of optically active SiV centers, as demonstrated in our earlier studies.^8,33^ Consequently, subsequent overgrowth of the porous diamond film with a Si-doped diamond layer is necessary to incorporate the SiV centers.

Figure 1c,d shows SEM images of the initially porous diamond film overgrown with a thin diamond film containing SiV centers. The final diamond film still remains highly porous, with only a slight smoothing of the fine dendritic morphology. Thus, the BU approach results in a core–shell-like porous structure, where the core consists of intrinsic diamond and the shell comprises the SiV-containing diamond film, as later confirmed by PL measurements. Importantly, the final dendrite-like porous diamond structure is brittle, making it suitable for mechanical disintegration into individual NDs.

**Figure 2:**
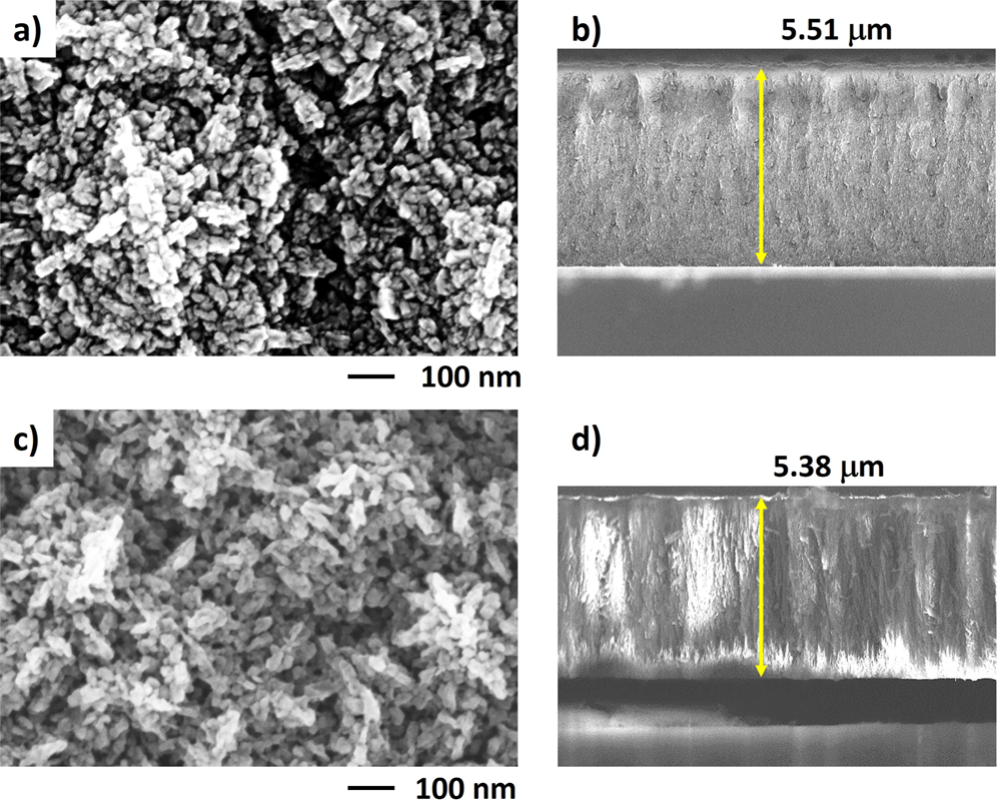
Surface morphology of the TD sample—top and cross-sectional SEM images of the initial NCD film (a,b) and MSTE-treated NCD film (c,d).

Representative top- and cross-sectional SEM images of the initial and MSTE-treated NCD diamond films (TD approach) are depicted in Figure 2. The surface morphology of the initial SiV-containing diamond film (Figure 2a) reveals nanosized diamond crystals, predominantly ranging from 20 to 50 nm, which are homogeneously distributed throughout the entire thickness (Figure 2b).

The initial thickness of the NCD film is approximately 5.5 μm. After the MSTE treatment, which involves annealing in air at 450 °C for 5 h with LiNO_3_ (Figure 2c,d), the film exhibits a nanoporous character with a slightly reduced thickness to approximately 5.4 μm. During the MSTE processes, nondiamond sp^1^-hybridized (*trans*-polyacetylene) and sp^2^-hybridized carbon phases (amorphous carbon and graphitic carbon) are preferentially etched away due to higher reactivity with oxygen species (NO_2_, O_2_) compared to sp^3^-hybridized diamond.^34^ Since these are predominantly localized at the grain boundaries, a porous diamond matrix is formed throughout the entire film thickness (Figure 2d).

The presence of the molten LiNO_3_ salt on the diamond surface affects the chemical reactions and eliminates the amount of sp^1^ and sp^2^ bonded carbon phases by the following set of reactions^35,36^
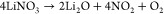
1
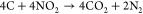
2
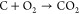
3

This series of chemical reactions leads to uniform selective etching of sp^1^- and sp^2^-hybridized carbons at 450 °C. The resulting porous diamond film is sufficiently brittle, with weakened intercrystalline bonds, allowing it to be easily transformed into the sub-100 nm fragments of NDs. Since the SiV centers are already embedded in the diamond crystals, the TD approach does not require any additional overgrowth, unlike the BU approach.

### Raman Spectroscopy and Photoluminescence of BU and TD Porous Diamond Films

To evaluate the material quality of the prepared diamond films and the PL properties of SiV centers, Raman spectroscopy and PL measurements were conducted.

**Figure 3:**
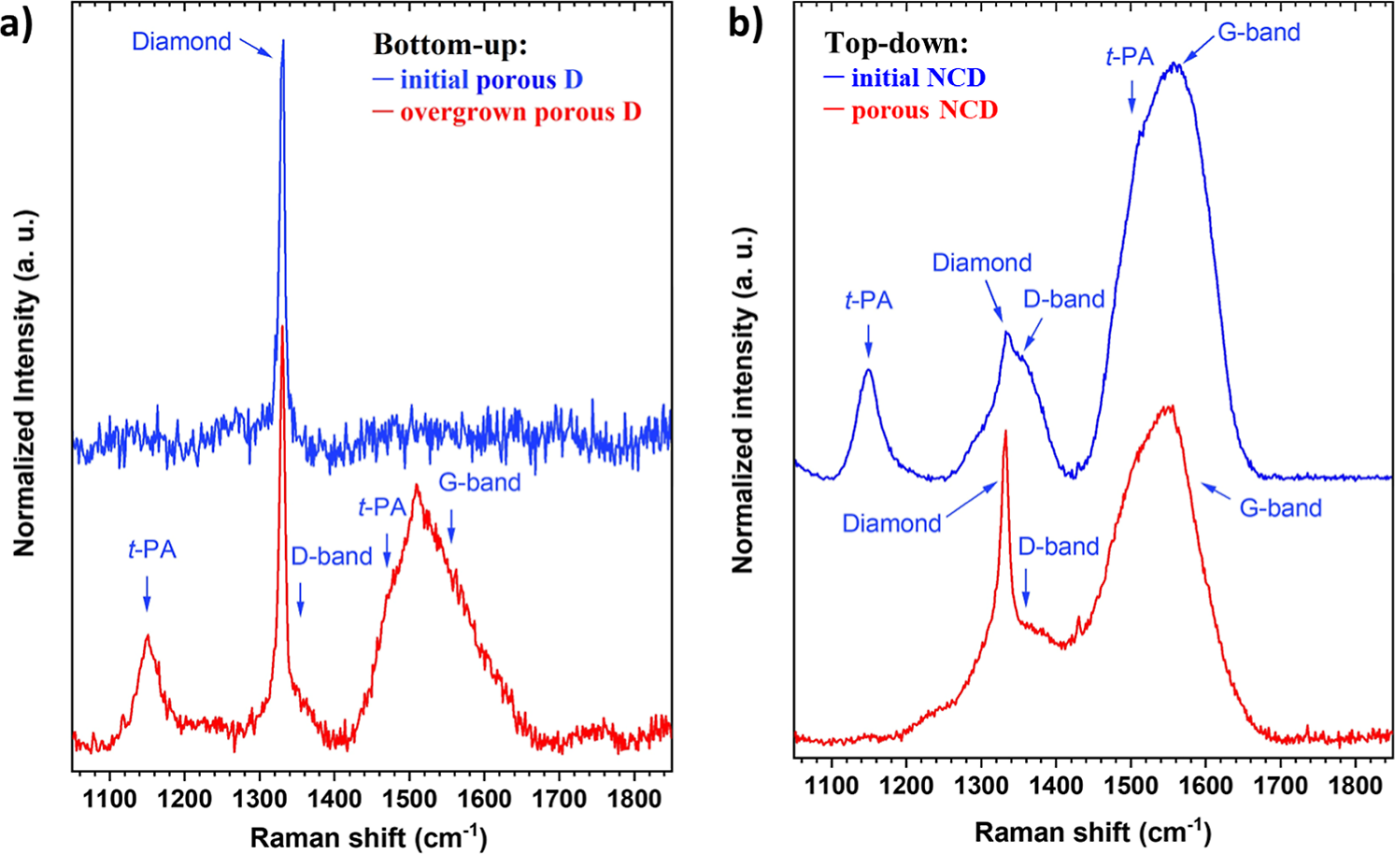
(a) Raman spectra of the BU initial porous diamond film without SiV centers (blue) and the overgrown porous diamond film with SiV centers (red). (b) Raman spectra of the TD initial NCD film (blue) and the MSTE-treated diamond film (red) (442 nm line excitation laser).

Figure 3a displays the Raman spectra of the initial porous and Si-doped overgrown porous diamond films (BU approach). The spectrum of the initial porous film (blue) shows only a single sharp peak centered at 1330 cm^–1^, corresponding to the zone center mode of the sp^3^ carbon phase. This confirms excellent diamond phase purity specific to the CO_2_-rich growth regime.^32^ In contrast, the Raman spectrum of the overgrown Si-doped diamond film (red) reveals additional bands, although it is still dominated by a narrow and intense peak related to the diamond phase. Broad bands centered at approximately 1356 cm^–1^ (D band) and 1552 cm^–1^ (G band) are associated with sp^2^ carbon phases. Two other broad bands centered at 1153 and 1473 cm^–1^ correspond to the carbon–hydrogen bending and carbon–carbon stretching modes of *trans*-polyacetylene (*t*-PA) located at the grain boundaries.^37,38^ The former can be considered direct evidence of nanometer-sized diamond grains.

The higher quality of the primary porous diamond layer compared to the overgrown layer is attributed to the high oxygen content in the gas mixture, which effectively etches away the nondiamond carbon phases during the CVD growth. Consequently, the film is almost purely composed of sp^3^ phases, where surface defects act as the new nucleation centers, preventing the formation of large diamond crystals, and finally the crystal growth and resulting in a porous dendrite-like morphology.^25,39^

The top diamond layer containing SiV centers must be grown without oxygen content in the gas mixture, as oxygen suppresses the formation of optically active SiV centers.^33,40^ This inevitably leads to the formation of sp^2^ phases and CH_*x*_ species on the diamond surface, such as *trans*-polyacetylene chains (*t*-PA), as confirmed by Raman spectroscopy (Figure 3a, red line). Given that the scattering cross-section of sp^2^ bonds in the Raman spectrum excited by visible light is 50 times larger,^38^ the overgrown film can be still considered as a nanocrystalline film with a distinct diamond character.

Raman spectra of the TD sample before and after the MSTE treatment are shown in Figure 3b. The spectrum of the initial diamond film (blue) reveals relatively intense nondiamond carbon features, dominated by *t*-PA bands (1149 and 1508 cm^–1^) and the D and G bands (1352 and 1572 cm^–1^). A weak diamond peak is also resolved at 1331.5 cm^–1^.

After MSTE treatment (red), a significant reduction of nondiamond phases is observed, and the sharp diamond peak (1331.5 cm^–1^) becomes well-recognizable. The *t*-PA bands have completely disappeared, and the intensity of the D and G bands has significantly decreased, indicating selective removal of the nondiamond phases located at the grain boundaries. The G band now consists of two main bands: a peak around 1560 cm^–1^ attributed to the usual G_1_ mode of graphite due to the in-plane bond stretching motion of pairs of sp^2^ carbon atoms^41^ and a downshifted G_2_ band at 1534 cm^–1^ attributed to amorphous carbon.^42^

**Figure 4:**
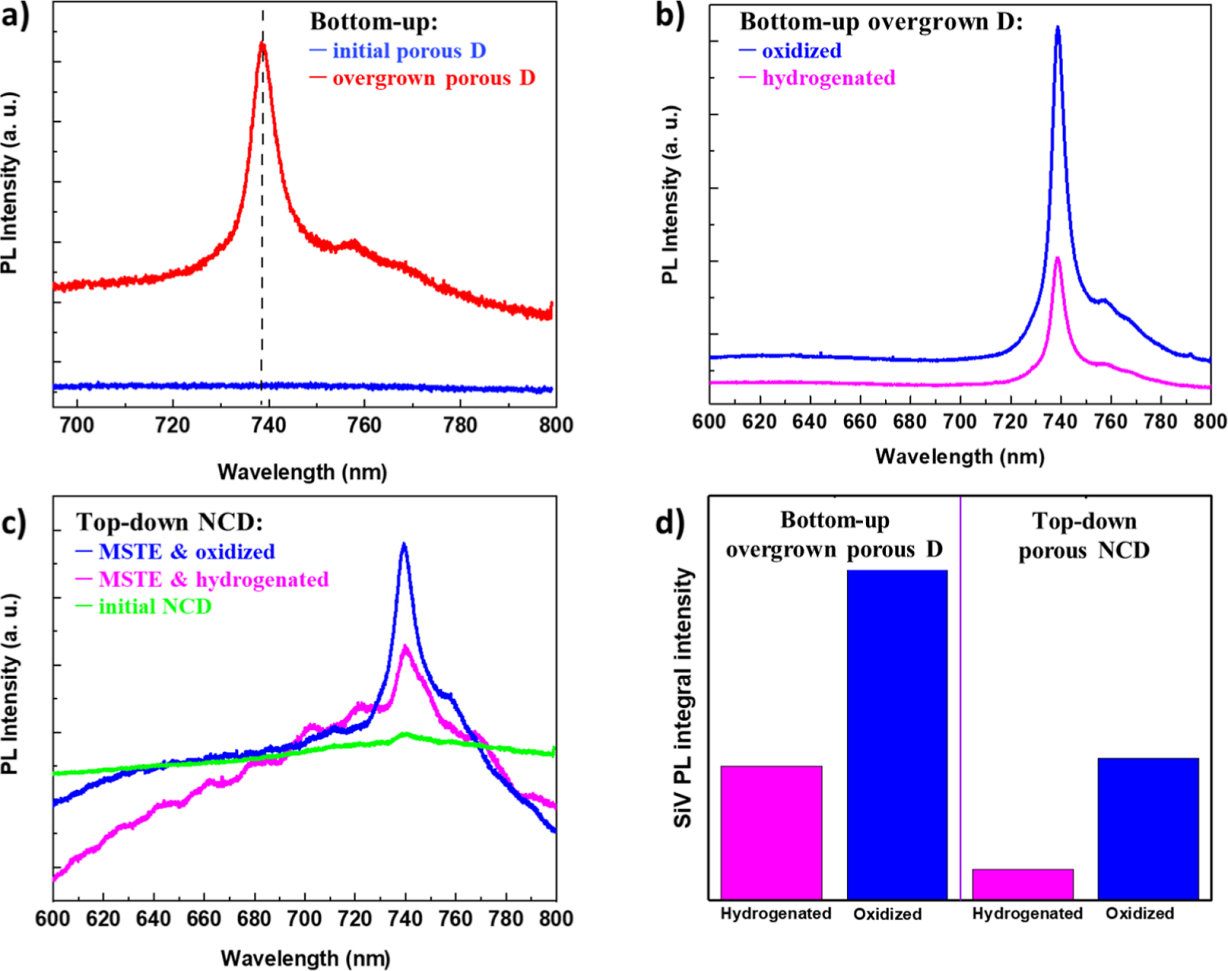
(a) PL spectra of the BU initial porous diamond film without SiV centers (blue) and the overgrown porous diamond film with SiV centers (red) and (b) overgrown porous diamond films with SiV centers after hydrogenation and oxidization of the diamond surface. PL spectra of TD initial and MSTE-treated NCD films after hydrogenation and oxidization of the diamond surface (c). Comparison of SiV integral intensities for porous diamond films prepared by BU (left) and TD (right) approaches (d) (442 nm line excitation laser).

PL spectra of BU and TD diamond films are shown in Figure 4. For the BU porous diamond layer grown at a high oxygen content, no PL signal was detected at the position of the SiV zero phonon line (ZPL) at 738.8 nm (Figure 4a, blue curve). This indicates that the primary BU porous sample is free of optically active SiV centers in the diamond layer as the result of the specific growth conditions discussed above.^33,40^ In contrast, the porous diamond layer overgrown in the focused MW plasma system reveals a strong SiV ZPL at 738.8 nm with a typical red-shifted sideband.^15,43^ This can be explained by the core–shell structure, where nonphotoluminescent diamond grains (core) are coated with a thin SiV-active diamond layer (shell).

We further investigated to what extent the SiV PL emission can be controlled by hydrogen or oxygen surface termination of a porous diamond layer using plasma treatment. As illustrated in Figure 4b,c, both hydrogenated and oxidized BU and TD samples exhibit enhanced SiV signals compared to the as-grown sample. Although hydrogenation does not completely suppress SiV PL, oxygen surface termination results in a 3-fold increase in SiV PL intensity relative to the H-terminated sample.

These findings are consistent with previous studies. Yang et al. reported a 1470-fold increase in PL intensity for SiV centers in diamond samples annealed in air at 700 °C for 20 min.^34^ This enhancement was attributed to a shift from nonradiative recombination (quenching for H-termination or sp^2^ carbon absorption) to radiative processes (O-termination, lower sp^2^ content). Such surface termination-induced PL enhancement requires optical centers to be predominantly located in close proximity to the diamond surface. Our earlier study^44^ demonstrated complete on/off switching of SiV PL by O-/H-termination for diamond films thinner than 10 nm. The incomplete switching observed in the present BU sample suggests that the top overgrown diamond layer containing optically active SiV centers exceeds 10 nm in thickness.

In contrast to the BU porous diamond overgrown with a SiV-containing diamond film, surface O-/H-termination has a weaker effect on TD diamond films. This may be due to a more uniform distribution of SiV centers throughout the diamond crystal volume or additional factors such as different growth regimes of diamond nanocrystals during the renucleation or variations in light absorptions between the BU and TD porous, etc. Nevertheless, it is noteworthy that despite the different BU and TD approaches, the PL emission behavior exhibits a similar surface-termination sensitivity trend.

SEM, Raman, and PL measurements indicate that the BU approach is more suitable for fabricating SiV-containing porous diamond layers, as overgrown porous crystals demonstrate superior quality and purity and provide a more favorable platform for the effective incorporation of optically active SiV centers. Furthermore, the morphology suppresses competitive nonradiative energy transfer across grain boundaries. These differences are reflected in the SiV ZPL peak parameters (maximum position and FWHM) between the BU and TD samples (see Table 1). We conclude that variations in the size of the optically active regions of diamond crystals and the magnitude of induced stress in the diamond film contribute to these differences.^45^

**Table 1:** SiV ZPL Data for BU and TD Porous Structures Terminated by H_2_ and O_2_ Plasma^a^

approach	termination	FWHM (nm)	SiV ZPL (nm)
BU	H_2_ plasma	6.2	738.8
	O_2_ plasma	6.1	739.0
	nanodiamonds^b^	7.3	738.7
TD	H_2_ plasma	7.3	739.6
	O_2_ plasma	8.5	739.5
	nanodiamonds^b^	12.7	739.8

a Data for fabricated BU/TD NDs are also added^.^

b Nanodiamonds produced from O-terminated porous films.

### Size, Structure, and Yield of PL-Emitting Nanodiamonds

**Figure 5:**
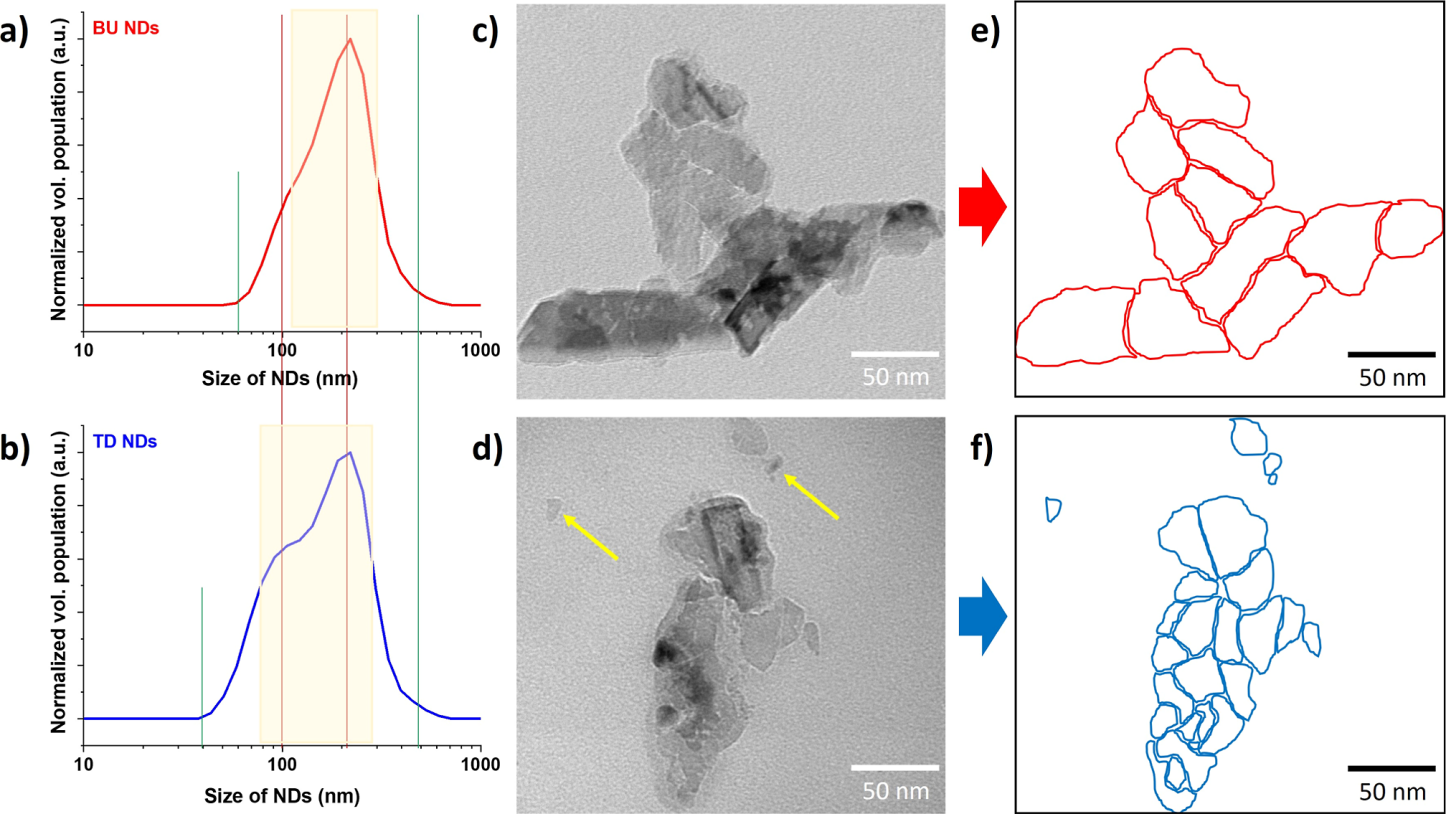
DLS size volume distribution (a,b), corresponding representative TEM images (c,d), and schematic visualization of individual SiV NDs in a cluster (e,f) of SiV NDs produced by BU (a,c,e) and TD (b,d,f) approaches.

In the subsequent technological stage, we disintegrated the oxidized porous BU and TD samples into individual NDs. A simple sonication technique was employed as a common method to disperse NDs in liquids and disaggregate them due to weakened intercrystallite bonds.^46,47^ The volumetric size distributions of the fabricated NDs from the BU and TD samples, as determined by DLS, are plotted in Figure 5a,b, respectively. For the BU sample, the volumetric size distribution (Figure 5a) ranges from 60 to 500 nm (indicated by the green lines), with the dominant fraction between 120 and 300 nm (yellow rectangle) and a maximum intensity at 220 nm (brown line).

TEM analysis (Figure 5c) reveals a representative ND cluster with an elongated dendrite-like shape, comprising smaller individual NDs (50 nm) and larger NDs with a total length of 230 nm. These observations corroborate the SEM images of the SiV-overgrown BU porous diamond layer shown in Figure 1, suggesting that the ND cluster resulted from disintegration of the porous precursor layer. The average zeta-potential (ζ) for SiV-NDs produced by the BU process is −24.6 mV as a result of the final treatment of the BU diamond film in the oxygen plasma.

For the TD sample, the volumetric size distribution (Figure 5b) extends from 40 to 500 nm with the dominant faction between 75 and 280 nm (yellow rectangle) and a maximum intensity at 220 nm (brown line). The volume distribution around 100 nm is notably higher for the TD sample compared to the BU sample.

TEM analysis (Figure 5d) shows a representative 200 × 100 nm cluster composed of 10–40 nm individual NDs. Smaller fragments and individual NDs (indicated by yellow arrows in Figure 5d) were also identified. The zeta-potential of SiV-NDs produced by the TD approach is −32.9 mV, resulting from their oxidized surface after MSTE and plasma treatment.^48^

It is clear that the disintegration into the individual NDs is not complete in both cases, and this step needs further optimization. Along with the optimization of the current sonication protocol parameters (time, power, and volume) and/or employment of the centrifugation for isolation of the sub-100 nm fraction, more energetic means might be used to obtain a fraction enriched in the individual SiV NDs or their sub-100 nm clusters. Compared to simple sonication, techniques like salt-assisted ultrasonic deaggregation^49^ or zirconia bead milling^50^ can be envisioned for a further shift of the size distribution to smaller size values. Another important point to discuss is the colloidal stability and the achieved zeta potential values. In both BU and TD cases, the zeta potential of the resulting SiV NDs is negative and close to or below −30 mV which is considered as the value ensuring long-term stability. Indeed, both samples remain colloidally stable for more than 1 year, yet sedimentation of the larger fraction naturally occurs. Recently, new approaches to ND stabilization in biological environments have emerged, often based on changing the stabilization mechanism from electrostatic to steric stabilization.^51,52^

**Figure 6:**
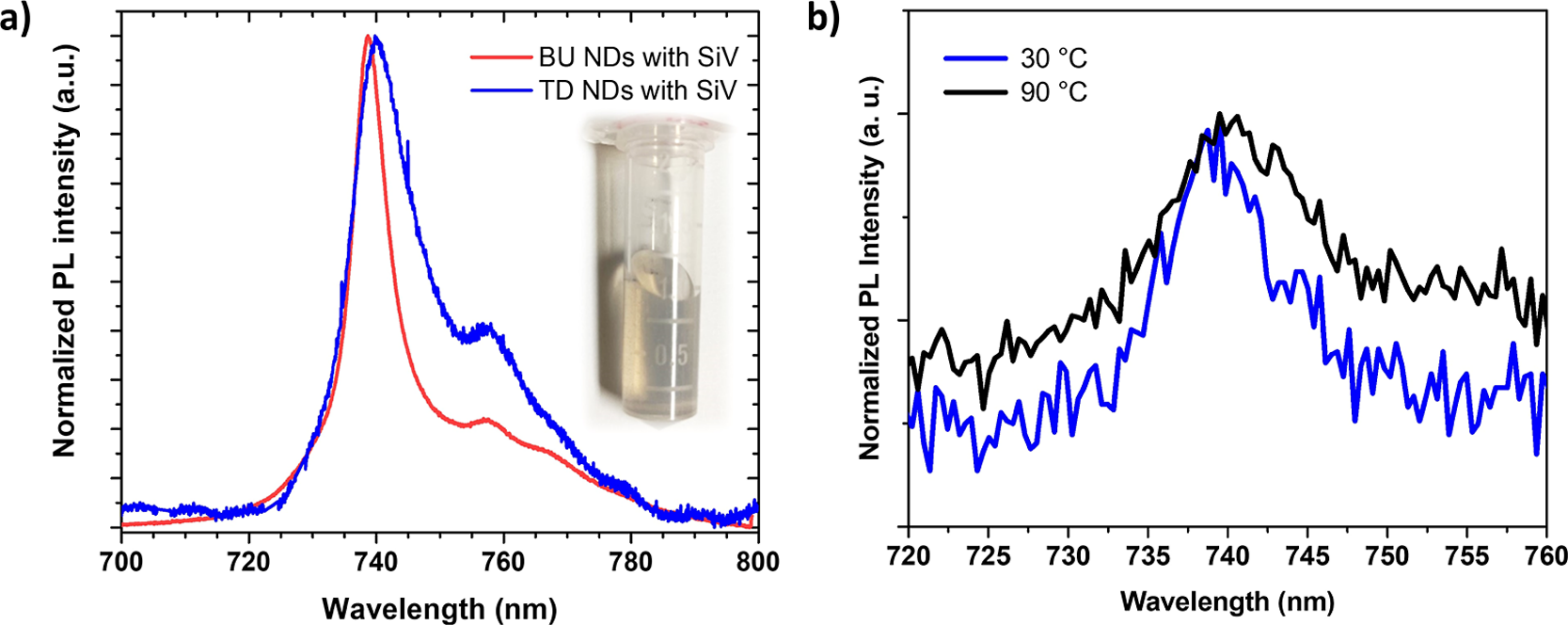
(a) Normalized PL spectra of SiV NDs fabricated using BU and TD approaches (442 nm line excitation laser). Inset–photograph of TD SiV NDs dispersed in DI water. (b) Normalized PL spectra of SiV NDs fabricated using TD measured at 30 °C (blue) and 90 °C (black) (638 nm excitation laser).

The successful fabrication of SiV light-emitting colloidally stable NDs using both BU and TD approaches is demonstrated in Figure 6a by a clear SiV PL emission, together with a photograph of TD SiV NDs dispersed in DI water shown in the inset. Please note a characteristic brownish hue that is characteristic for well-dispersed sub-100 nm NDs.

PL spectra measured from dried SiV ND deposits exhibit dominant SiV ZPL maxima for both NDs types, with line shapes comparable to those of the porous diamond films (Figure 4), confirming that the disaggregation treatment did not adversely affect the SiV PL parameters. Notably, BU-produced NDs display ZPL position at lower values by approximately 0.6 nm (Table 1), which can be attributed to the different morphology (core–shell) and released strain of the overgrown SiV NDs.^53^ TD SiV NDs also exhibit larger FWHM values, potentially due to the more defective character of constituting diamond nanocrystals (crystallographic defects and nitrogen-related stress resulting from the high nitrogen content in the gas mixture). Figure 6b shows a shift in the SiV ZPL maxima of normalized PL spectra of SiV NDs fabricated using TD due to the temperature increase from 30 °C (blue) to 90 °C (black), which represents an important prerequisite for the temperature sensing as elaborated further below.

These observations provide a direct proof-of-concept for the successful fabrication of optically active SiV NDs employing both BU and TD approaches. A key technological advance of both methods is their technological simplicity eliminating the need for additional steps commonly required in other techniques (i.e., neutron irradiation, ion implantation, annealing, etc.).^14,54^

The sensitivity of color centers in diamonds to temperature changes makes them suitable for temperature measurement. Our samples are particularly interesting in this aspect as they provide the platform for nanothermometry, e.g., in living cells.^55^ To demonstrate this functionality, we performed spectral measurements (including time-resolved) for a wide range of temperatures (from 30 to 100 °C) using a 638 nm excitation laser. The NDs are deposited on a heat-conductive silicon substrate for temperature-resolved spectroscopy. Since the shift in the ZPL and line width broadening are the main parameters that we extracted at different temperatures, a small number of NDs per focal volume is used (10 ± 2 NDs) to avoid agglomeration-induced broadening due to the different size of the NDs and other quantum electrodynamics effects.^56^ The ZPL position shows a red shift and spectral broadening as the temperature increases, see Figure 6b for 30 and 90 °C. This behavior is mainly attributed to the thermal lattice expansion that reduces the orbital overlap in bonds.^57^ An excitation wavelength of 638 nm is preferred due to the reduction of the background signal associated with NV and other color centers. Under excitation with a 532 nm laser line, NDs indeed demonstrate the presence of NV^0^ color centers due to the nitrogen background in the CVD mixture as mentioned above (Figure S1).

**Figure 7:**
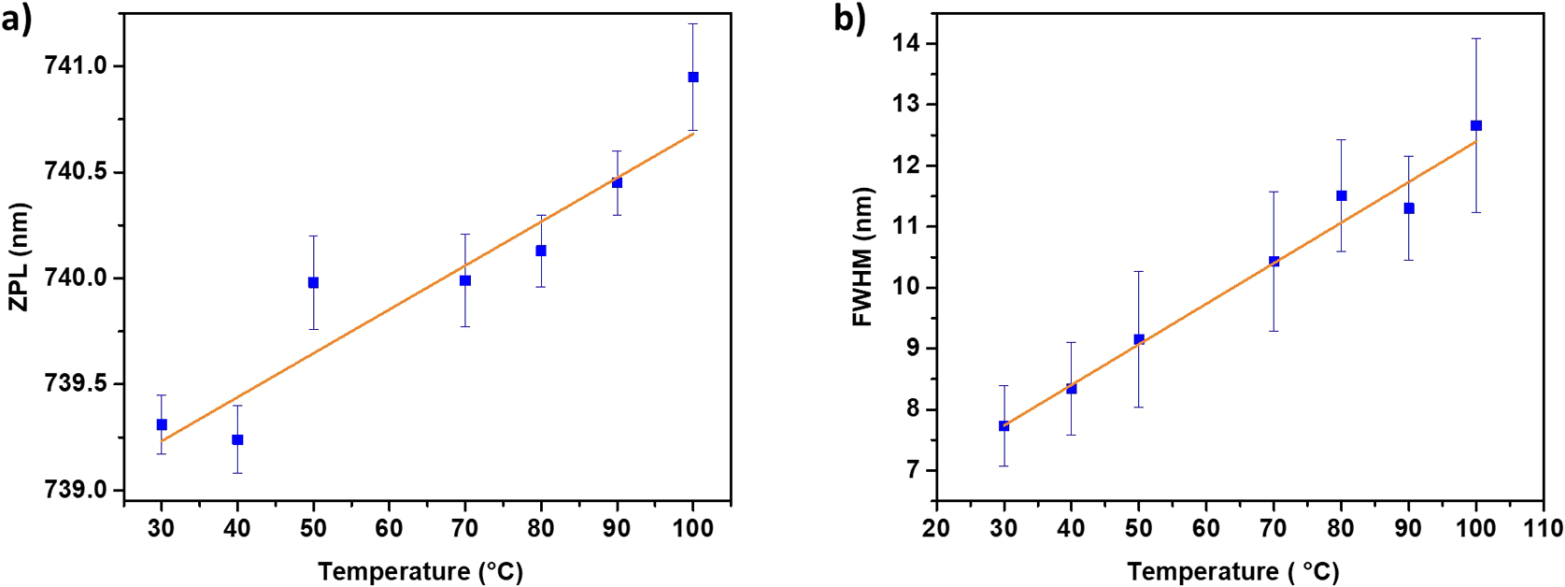
Temperature dependence of ZPL position (a) and of FWHM (b) for SiV NDs fabricated by the TD approach (638 nm excitation laser).

The ZPL shows a linear spectral red shift with a 0.022 ± 0.003 nm/K sensitivity, as shown in Figure 7a, while the line width exhibits a spectral broadening of 0.068 ± 0.004 nm/K (Figure 7b). Previous works have mainly reported spectral changes in smaller temperature ranges. For instance, a spectral red shift of 0.011 ± 0.002 nm/K and a halfwidth broadening of 0.011 ± 0.002 nm/K were reported using detonation NDs (for the temperature range of 22–40.5 °C)^58^ and 0.011 ± 0.013 nm/K spectral shift for HPHT fabricated NDs (for the temperature range of 25–37.5 °C).^55^ Recent reports on bulk diamonds show a spectral shift of 0.0124 ± 0.0002 nm/K (for a temperature range of 15–29 °C)^59^ and 0.016 ± 0.003 nm/K (for a temperature range of 20–90 °C).^60^ The difference in the spectral response as compared to previous work is attributed to the difference in the host composition and its surface chemistry in addition to the temperature range. As shown in work by Lagomarsino et al.,^61^ the change in the ZPL spectral position as a function of temperature depends on the choice of temperature window. Time resolved measurements using time-correlated single-photon counter show that the excited-state lifetime remains the same in the measured temperature ranges (20–90 °C), see Figure S2.

Finally, an important parameter in the fabrication of SiV NDs is the obtainable amount, i.e., production yield. For instance, the HPHT fabrication of NDs containing NV centers yields an estimated 10%.^62,63^ The effective production of SiV NDs has been intensively studied by using both HPHT and CVD techniques. The HPHT process (BU approach, direct ND synthesis from organic precursors) has demonstrated reliability in producing tens of mg of NDs in size to 10 nm in one run in size up to 10 nm with high crystallographic quality and bright SiV PL.^17,63,64^ There are still many technological challenges that prevent the process from becoming more widespread. For example, the HPHT devices are very expensive and often custom-built, and the correct design of the reaction zone, ensuring a homogeneous distribution of heat and pressure, is also very demanding^65^

MW plasma CVD processes relying on the self-doping of NDs from the silicon substrate are commonly used as the simplest methods. Li et al. reported narrow PL line width CVD-grown NDs with SiV centers.^66^ Recently, Bogdanov et al. introduced a dual-doping process for fabricating SiV and GeV-doped NDs.^67^ However, such a CVD fabrication typically yields only a few >100 nm NDs per run. Further CVD technological progress applying a multilayer-like compartment was presented by Dallaire et al.^21^ This process was subsequently optimized by Feudis et al., who achieved a production yield of a few mg NDs per CVD run.^22^ In contrast to the BU approaches (i.e., HPHT and CVD processes), ball milling has been applied as the TD approach. However, only a proof-of-concept level has been achieved due to limitations such as the large consumption of cleaning chemicals (mainly acids) and the long-term treatment needed for ND purification.^20^

In this work, we implemented a multilayer-like compartment strategy similar to that of Dallaire et al.,^21^ yet unique technological steps were developed for the fabrication of SiV porous diamond layers using either the BU or TD approach. Our approaches can yield 22 mg for BU (1 μm thick diamond on 4 in. with 20% weight losses) and 28 mg for TD (6 μm thick film on 2 in. with 30% weight losses) in one run, considering 100% conversion of the porous diamond layers to NDs.

The initial thicknesses of the grown diamond layers were selected as a compromise solution for fundamentally different MW plasma CVD systems. The BU approach utilized a linear antenna MW plasma system, characterized by slow diamond growth (approximately 150 nm/h) over large areas (at least 4 in.) at low pressure (<10 mbar). In contrast, the TD approach employed a focused MW plasma system, featuring rapid growth (3 ÷ 5 μm/h) over a limited deposition area (2 in.) at medium pressure (90 ÷ 200 mbar). Furthermore, the BU samples exhibit a brittle nature due to their porous structure, facilitating easy fragmentation into individual NDs via ultrasonic treatment. However, the required overgrowth with SiV-incorporated diamond necessitates a limitation on the thickness of the porous layer. Conversely, the TD samples are not as constrained by the initial film thickness owing to the employed molten-salt thermal etching for porous structure fabrication. Nevertheless, considerations must be made to efficiently and uniformly disintegrate the thicker nanoporous layer in TD samples compared to the thinner BU counterparts.

In the context of SiV NDs, the BU core–shell approach demonstrates significant potential for fabricating high-quality surface-fluorescent NDs owing to the sharp and narrow PL line of SiV centers. However, further technological improvements are still needed to directly grow doped porous films using the linear antenna CVD technique. The TD approach offers a distinct advantage in terms of scalability, as it enables the production of hundreds of milligrams of NDs with relative ease. This is primarily due to the simplicity of NCD growth and the applicability of the MSTE process to thick films (>50 μm). Extending this concept, we have successfully produced porous boron-doped NCD films^30^ and boron-doped NDs using the TD approach. This demonstrates the versatility of the presented technological approaches, which can be broadly applied to produce NDs with various dopants and properties.

These methodologies offer a promising avenue for the large-scale production of functionalized NDs, potentially meeting the growing demand for these materials in diverse applications such as quantum sensing, bioimaging, and nanoscale electronics.

## Conclusions

This study demonstrates two CVD-based approaches for fabricating SiV-containing NDs: BU and TD. Both methods successfully produced nanoporous diamond layers with bright SiV PL, yielding NDs ranging from 40 to 500 nm with stable SiV emission. The BU approach generated high-quality, dendrite-like porous core–shell-like structures, while the TD approach created uniform nanoporous structures with efficient nondiamond phase removal. SiV emission in these BU/TD nanoporous structures revealed a sensitivity to surface terminations. The TD-fabricated SiV NDs exhibited potential for nanothermometry applications with a ZPL sensitivity of 0.022 ± 0.003 nm/K.

The presented CVD-based approaches offer scalable and cost-attractive manufacturing solutions to existing ND’s fabrication techniques, including a streamlined production at high-yield with tailored properties. These approaches also provide promising routes for further development of functional nanoscale diamond structures, addressing the growing demand for quantum sensing, bioimaging, and optoelectronic applications. Future work should focus on optimizing growth parameters and exploring additional dopants to further expand the utility of these NDs.

## Data Availability

The data are available at: https://zenodo.org/records/10.5281/zenodo.11065976.

## Supporting Information Available

CVD process parameter tables for each step of the growth and termination process, excitation laser versus signal-to-noise ratios, and excited-state lifetimes spectra (PDF)
