# Supplementary material for: High-Yield Production of SiV-Doped Nanodiamonds for Spectroscopy and Sensing Applications
Source: ACS Appl Nano Mater. 2024 Oct 25;7(21):24766–77. doi: 10.1021/acsanm.4c04676 (PMC11555635; doi:10.1021/acsanm.4c04676)
Supplement: Supplementary file 1 — an4c04676_si_001.pdf [file an4c04676_si_001.pdf]

Supporting Information

# High-Yield Production of SiV-Doped Nanodiamonds for Spectroscopy and Sensing Applications

Alexander Kromka[a*], Marián Varga[a,b], Kateřina Aubrechtová Dragounová[a,c], Oleg Babčenko[a], Rene Pfeifer[a], Assegid M. Flatae[d], Florian Sledz[d], Farzana Akther[d], Mario Agio[d,e], Štěpán Potocký[a], and Štěpán Stehlík[a]

[a] *Institute of Physics, Czech Academy of Sciences, Cukrovarnická 10/112, Prague 6, 162 00, Czech Republic*
[b] *Institute of Electrical Engineering, Slovak Academy of Sciences, Dúbravská cesta 9, Bratislava, 841 04, Slovakia*
[c] *Faculty of Nuclear Sciences and Physical Engineering, Czech Technical University in Prague, Břehová 7, 115 19, Prague 1, Czech Republic*
[d] *Laboratory of Nano-Optics and Cμ, University of Siegen, Walter-Flex-Str. 3, 57072 Siegen, Germany*
[e]*National Institute of Optics (INO-CNR), Largo Enrico Fermi 6, 50125 Florence, Italy*
[*]Email: kromka@fzu.cz

**Table S1:** CVD process parameters used for the growth of the initial porous diamond film without SiV in a linear antenna pulsed microwave plasma system.

| **BU (no SiV)** | **$CH_4$ (sccm)** | **$CO_2$ (sccm)** | **$H_2$ (sccm)** | **$T_S$ (°C)** | **$P_{MW}$ (kW)** | **p (Pa)** | **t (h)** |
|---|---|---|---|---|---|---|---|
| ***Starting layer*** | 5 | 30 | 100 | 380±5 | 2x2 | 15 | 2 |
| ***Porous layer*** | 5 | 20 | 25 | 380±5 | 2x2 | 15 | 15 |

*$T_S$ – substrate temperature, $P_{MW}$ – provided microwave power (the sources are from the two sides), p – gas mixture pressure, t – deposition duration*

**Table S2:** CVD process parameters used for the growth of Si-doped diamond top-layer in a focused microwave plasma system.

| **BU (with SiV)** | **$CH_4$ (sccm)** | **$H_2$ (sccm)** | **$T_S$ (°C)** | **$P_{MW}$ (kW)** | **p (kPa)** | **t (h)** |
|---|---|---|---|---|---|---|
| ***Top-layer with SiV*** | 3 | 300 | 680±20 | 3 | 6 | 0.2 |

*$T_S$ – substrate temperature, $P_{MW}$ – provided microwave power, p – gas mixture pressure, t – deposition duration*

**Table S3:** CVD process parameters used for the growth of thick NCD film with SiV in a focused microwave plasma system.

| **TD** | **$CH_4$ (sccm)** | **$N_2$ (sccm)** | **$H_2$ (sccm)** | **$T_S$ (°C)** | **$P_{MW}$ (kW)** | **p (kPa)** | **t (h)** |
|---|---|---|---|---|---|---|---|
| ***NCD with SiV*** | 9 | 4.5 | 300 | 880±20 | 4 | 9 | 4 |

*$T_S$ – substrate temperature, $P_{MW}$ – provided microwave power, p – gas mixture pressure, t – deposition duration*

**Table S4:** Process parameters for hydrogen termination of BU and TD porous diamond samples in focused microwave plasma system.

| Termination | $H_2$ (sccm) | $T_S$ (°C) | $P_{MW}$ (kW) | p (kPa) | t (min) |
|---|---|---|---|---|---|
| **$H_2$ plasma** | 300 | 650±20 | 3 | 6 | 10 |

*$T_S$ – substrate temperature, $P_{MW}$ – provided microwave power, p – gas pressure, t – process duration*

**Table S5:** Process parameters for oxygen termination of BU and TD porous diamond samples in RF plasma system.

| Termination | $O_2$ (sccm) | $T_S$ (°C) | $P_{RF}$ (W) | p (Pa) | t (min) |
|---|---|---|---|---|---|
| **$O_2$ plasma** | 50 | <100 | 100 | 62 | 2 |

*$T_S$ – estimated temperature, $P_{RF}$ – provided radiofrequency power, p – gas pressure, t – process duration*

## Excitation laser vs signal-to-noise ratio

The signal-to-noise ratio (SNR) of SiV color centers in NDs depends on the excitation laser. Excitation using 532 nm laser leads to nitrogen-vacancy and another radiative background that deteriorates the signal strength of SiV color centers, see Fig. S1a. The first peak at 532 nm is the excitation laser, the two double peaks around 575 nm correspond to the Raman and $NV^0$ ZPL, while the small peak at 738 nm corresponds to SiV color centers. Since $NV^0$ is spectrally broad and can even overlap with the diamond Raman line at high temperatures, it is not used as a sensor in this work. However, it is clear that the $NV^0$ has a wide phonon wing that can reach to the spectral window of the SiV color centers, as the result it affects the signal-to-noise ratio of SiV color centers.

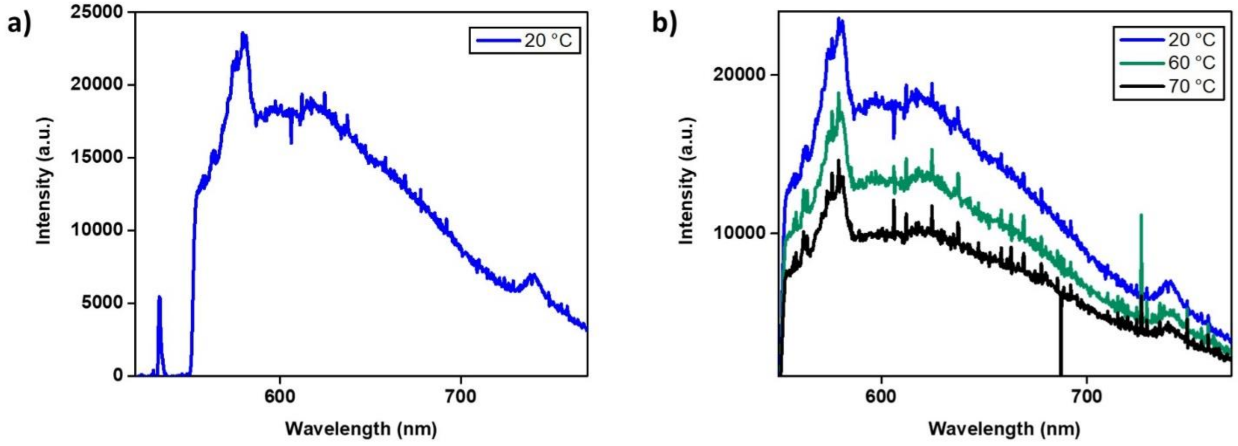


**Figure S1.** (a) NV- and SiV color centers using 532 nm excitation laser. (b) Spectral features of SiV and NV color centers at 20 °C, 60 °C and 70 °C.

Using 638 nm excitation laser, one can improve the SNR of SiV color centers as shown in Fig. S2. The signal around 696 nm corresponds to the diamond Raman line.

**Excited-state lifetime**

The excited-state lifetime (~0.8 ns) remains the same in the temperature range from 30-100 °C (Fig. S2). The measurements were performed using time-correlated-single-photon counter.

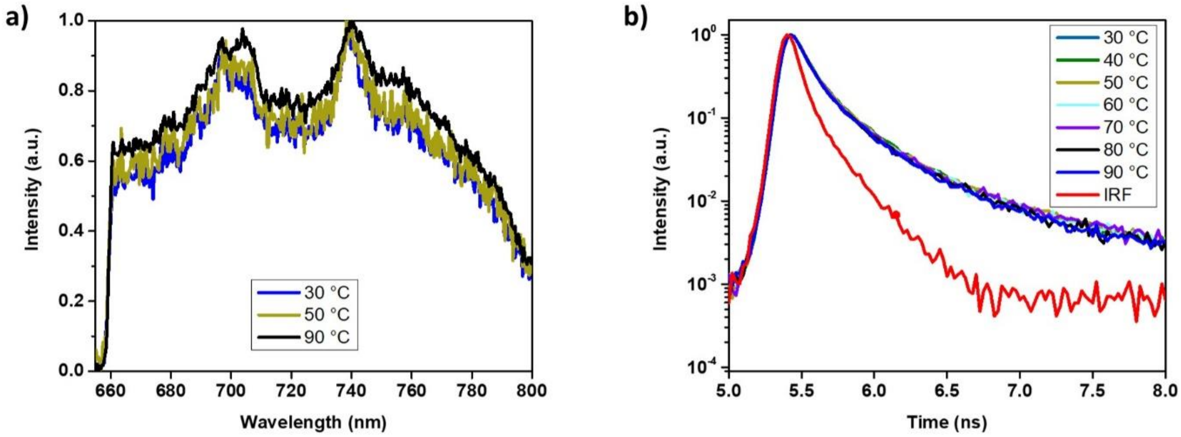


**Figure S2:** (a) Spectral features of SiV color centers excited using 638 nm at 30 °C, 50 °C and 90 °C. (b) Excited-state lifetime of SiV color centers at different temperatures.
